# 3D Shape Reconstruction of 3D Printed Transparent Microscopic Objects from Multiple Photographic Images Using Ultraviolet Illumination

**DOI:** 10.3390/mi9060261

**Published:** 2018-05-27

**Authors:** Keishi Koyama, Masayuki Takakura, Taichi Furukawa, Shoji Maruo

**Affiliations:** 1 Graduate School of Engineering, Yokohama National University, 79-5 Tokiwadai, Hodogaya, Yokohama 240-8501, Japan; koyama-keishi-gx@ynu.jp (K.K.); takakura-masayuki-vw@ynu.jp (M.T.); 2 Faculty of Engineering, Yokohama National University, 79-5 Tokiwadai, Hodogaya, Yokohama 240-8501, Japan; furukawa-taichi-xp@ynu.ac.jp

**Keywords:** 3D shape reconstruction, shape from silhouette, 3D printing, additive manufacturing, micro-stereolithography, transparent object, photopolymer

## Abstract

We propose and demonstrate a simple, low-cost, three-dimensional (3D) shape acquisition method for transparent 3D printed microscopic objects. Our method uses ultraviolet (UV) illumination to obtain high-contrast silhouette images of transparent 3D printed polymer objects. Multiple silhouette images taken from different viewpoints make it possible to reconstruct the 3D shape of this transparent object. A 3D shape acquisition system consisting of a UV light-emitting diode, charge-coupled device camera and a rotation stage was constructed and used to successfully reconstruct the 3D shape of a transparent bunny model produced using micro-stereolithography. In addition, 3D printed pillar array models, with different diameters on the order of several hundred micrometers, were reconstructed. This method will be a promising tool for the 3D shape reconstruction of transparent 3D objects on both the micro- and macro-scale by changing the imaging lens.

## 1. Introduction

In recent years, various kinds of 3D printing technologies, from macro- to micro-scale devices, have been developed and widely used with a wide variety of materials including polymers, metals and ceramics [1,2,3]. To use 3D printed parts for final products, techniques for measuring the 3D shape of a 3D printed part are indispensable. X-ray computed tomography (CT) has been utilized as a powerful 3D shape measurement tool for 3D printed parts to date [4]. Recently, it has also been used to measure microscopic 3D printed parts [5]. However, because X-ray CT equipment is very expensive, it is not suitable as a convenient method for evaluating the 3D printed parts produced by the low-cost desktop 3D printers used by educators, hobbyists and professional designers.

On the other hand, several optical measurement methods including photogrammetry, structured light, shape from shading and shape from silhouette (SFS) have been developed as inexpensive techniques for acquiring the shape of a 3D object [6,7,8,9,10]. The SFS method, in particular, is a simple way to reconstruct a 3D shape of the target 3D object using multiple silhouette images captured from several directions. It has an advantage because it can be realized using only small and inexpensive pieces of equipment such as a camera, lighting device and rotary stage. Furthermore, the use of a zoom lens makes it possible to measure small 3D objects as small as 1 mm or less [10]. Therefore, it can be expected to be used for measuring the 3D printed microscopic objects produced by micro-scale 3D printing techniques such as single-photon micro- and two-photon stereolithography [11,12]. However, the conventional SFS method is difficult to use to measure transparent 3D objects such as the products of stereolithography and material jetting using ultraviolet (UV) curable polymers, because the silhouette of the transparent object includes the light that passed through the interior of the object in addition to its actual contour.

To overcome the above limitation of the conventional SFS method using visible light, we propose a novel method to acquire the shape of transparent 3D printed parts using UV illumination in this study. Most of the transparent 3D polymer parts produced by stereolithography absorb little visible light but absorb UV light strongly. Thus, it is possible to obtain high-contrast silhouette images using UV illumination. For this reason, even transparent 3D printed parts can be evaluated using the SFS method with UV light. Although there are some alternative methods for this, including local heating using infrared light [13] and the polarization of the reflected and emitted light [14], our method has advantages including the ability to capture silhouette images without a background subtraction process and a relatively high resolution as a result of the use of UV light.

We constructed a simple, low-cost 3D shape acquisition system using a UV light-emitting diode (LED), a UV-sensitive charge-coupled device (CCD) camera and a motorized rotation stage to demonstrate the usefulness of our proposed method. Using the optical system, we acquired the 3D shape of a bunny model as a case study. In addition, the accuracy of the 3D shape acquisition was evaluated by measuring an array of pillars with diameters ranging from 100–350 μm.

## 2. Materials and Methods

### 2.1. 3D Shape Acquisition Based on the Shape from Silhouette (SFS) Method

**Figure 1:**
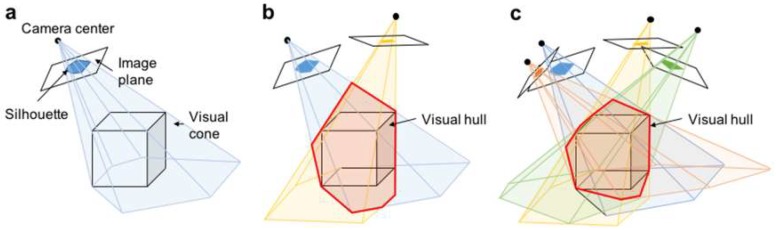
3D shape reconstruction based on shape from silhouette (SFS) method. (**a**) visual cone obtained by back projection of silhouette image; (**b**) visual hull obtained by intersection of two visual cones; and, (**c**) 3D shape acquired by bounding geometry of resultant visual hull with multiple visual cones.

The first step in reconstructing the 3D shape of a 3D printed object using the SFS method is to acquire silhouette images of the target object from various directions. In the standard SFS method, a silhouette image is obtained by calculating the difference between an input image that includes the target object and a previously captured background image. In contrast, our SFS method using transmitted UV light illumination does not require background subtraction processes to capture silhouette images, because the background surrounding the target object has a uniform brightness and its contrast is enough high to binarize the silhouette images. Then, as shown in Figure 1, the binarized silhouette image on the image plane is back projected to the camera center to obtain a visual cone that includes the target object (a cube in Figure 1). Next, multiple visual cones are obtained from different viewpoints by positioning the camera around the object or rotating the object using a rotating stage. Finally, the common part (visual hull) of the visual cones obtained from each viewpoint is calculated. In principle, because the target object exists inside the visual hull, this visual hull can be used to acquire the 3D shape of the target object [9].

Our method employs the strategy reported by Atsushi et al. [10] for acquiring a 3D shape from silhouette images. In this method, a voxel-based 3D model is first used to represent the 3D shape of the target object in the SFS method. Then, the voxel-based 3D model is converted to a triangular mesh model using the marching cubes algorithm [15]. This triangular mesh model can be easily imported by commercial CAD software.

### 2.2. 3D Shape Acquisition System Based on SFS with Ultraviolet Light

**Figure 2:**
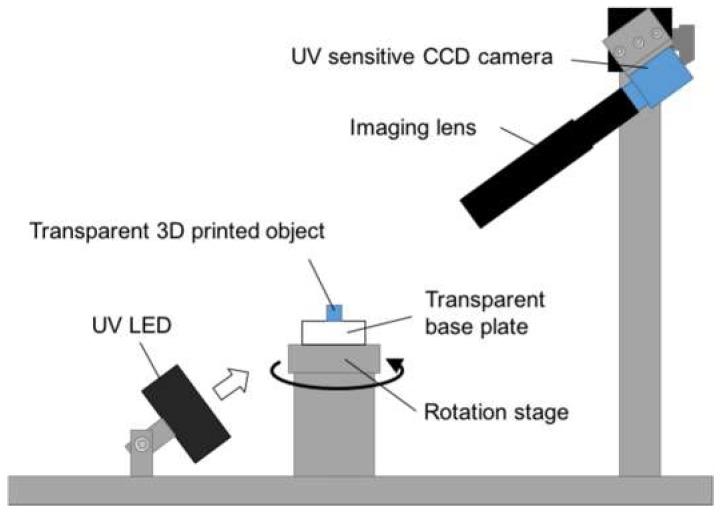
Optical setup for SFS using ultraviolet (UV) illumination. A silhouette image of the target object with UV illumination was captured by the UV sensitive charge-coupled device camera. Multiple silhouette images with different viewpoints were obtained by rotating the rotation stage at 10° intervals.

To obtain an accurate, high-contrast silhouette image of a transparent 3D microscopic object, we constructed a 3D shape acquisition system using a UV LED (MBRL-CUV7530-2, Moritex Corp., Saitama, Japan, light-emitting area: 30 × 75 mm, emission peak wavelength: 365 nm), an imaging lens (MML1-ST150, Moritex Corp., numerical aperture: 0.038, magnification: ×1, depth of field: 1.1 mm), a UV-sensitive CCD camera (XC-EU 50, Sony Corp., Tokyo, Japan) and a motorized rotation stage, as shown in Figure 2. The CCD camera is sensitive to light with wavelengths ranging from 300 nm to 420 nm. The silhouette images were captured at a resolution of 720 × 480 pixels. The CCD camera and attached lens were fixed at 60° angles from the horizontal plane. The rotation stage consisted of a stepping motor (AS 46 AAD, Oriental Motor Co., Ltd., Tokyo, Japan) and its controller (MSCTL 102, Suruga Seki Co., Ltd., Shizuoka, Japan).

To reconstruct the 3D shape from the acquired silhouette images, in the SFS method, it was necessary to calibrate the internal and external parameters of the camera. Our experiments used a calibration method developed by Lavest et al. [16] to determine parameters such as the distance between the camera and the object and the relationship between the acquired image and the real-world coordinates. A 2 mm square cube made by cutting was the target object used to calibrate the camera.

After camera calibration, a transparent 3D printed microscopic part was placed in the center of the rotation stage and its silhouette images from different viewpoints were captured by rotating the stage at 10° intervals. The 3D shape of the 3D printed part was reconstructed using the captured silhouette images with a reconstruction program developed by Atsushi et al. [10].

### 2.3. Micro-Stereolithography Systems and Photocurable Resins

In our experiments, two types of laboratory-made micro-stereolithography systems were used to make 3D printed micro-parts. One was a bottom-up system (free-surface method) based on single-photon polymerization using a He-Cd laser (IK5551R-F, Kimmon Koha Co., Ltd., Tokyo, Japan, wavelength: 325 nm). The laser spot size of this system is approximately 12 µm. The minimum layer thickness for 3D printing is 30 µm because of the viscosity of the resin. Therefore, this laser was suitable for making millimeter-sized 3D objects such as microchannels, scaffolds and energy harvesters [11,17,18]. Therefore, this system was used to make a bunny model (size: 1.2 × 0.8 × 1.1 mm) using a commercial epoxy-based photocurable resin (TSR-883, CMET Inc., Yokohama, Japan) as an example of a transparent complex 3D object. The other laboratory-made micro-stereolithography system used was a top-down system (constrained-surface method) based on single-photon polymerization using a blue laser diode (Cobolt 06-MLD, Cobolt AB, Solna, Sweden, wavelength: 405 nm). In this system, the blue laser beam is collimated and introduced into a Galvano scanner (GM-1010, Canon Inc., Tokyo, Japan) and focused by an objective lens (numerical aperture: 0.1). Both the focal spot size and minimum layer thickness of this system are 5 µm. Therefore, this system was used to create finer structures with higher resolutions compared to those created by the bottom-up system. The resin used in the bottom-up system is not suitable for the system using a blue laser beam because of its absorption spectrum. Therefore, we prepared a laboratory-made photocurable resin containing an acrylate monomer (SR399, Sartomer Inc., Exton, PA, USA, 95.1 wt %), a photoinitiator (Diphenyl(2,4,6-trimethylbenzoyl)phosphine oxide, Sigma-Aldrich, St. Louis, MO, USA, 1.0 wt %), an inhibitor (2-tert-Butyl-4-methylphenol, Sigma-Aldrich, St. Louis, MO, USA, 2.9 wt %) and a UV absorber (2-(5-Chloro-2-benzotriazolyl)-6-tert-butyl-p-cresol, Tokyo Chemical Industry Co. Ltd., Tokyo, Japan, 1.0 wt %). Using the top-down system with the laboratory-made acrylate-based photocurable resin, we fabricated a pillar array model that had four pillars with different diameters.

## 3. Results and Discussion

### 3.1. Transmission Spectrum of Photopolymer

**Figure 3:**
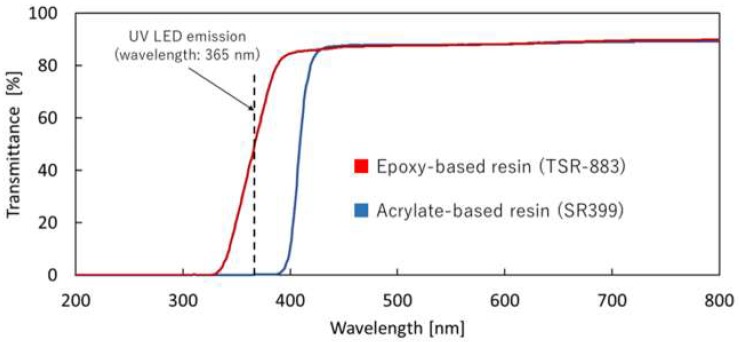
Transmission spectra of two cured resins: a commercial epoxy-based resin (TSR-883) and a laboratory-made acrylate-based resin containing SR399.

To measure the transmittance values of the two kinds of cured resins, thin films with a thickness of approximately 200 μm were prepared by curing both resins with a UV lamp. The transmission spectra of the thin films were measured using a UV-visible spectrometer (UV-1700, SHIMADZU Corp., Kyoto, Japan) (Figure 3). The transmittance values of the acrylate-based and epoxy-based resins at the UV LED emission peak wavelength (365 nm) were 0.1% and 46.3%, respectively. Therefore, the acrylate-based resin was considered suitable for obtaining high-contrast silhouette images using the UV LED. Although the UV absorption of the epoxy-based resin was lower than that of the acrylate-based resin, it could also be used to obtain a sufficient number of high-contrast silhouette images after proper binarization without a background subtraction process, as shown in the following experiments.

### 3.2. 3D Shape Acquisition of Transparent 3D Printed Objects

**Figure 4:**
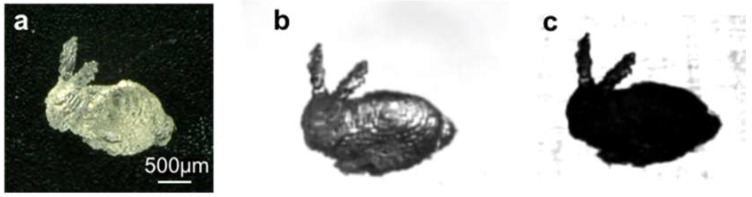
Comparison of silhouette images of a 3D printed bunny model using visible and UV light. (**a**) optical microscope image of an epoxy-based resin model of a bunny, produced using micro-stereolithography; (**b**) silhouette image obtained with visible transmitted light; and, (**c**) silhouette image obtained with UV transmitted light.

To demonstrate the usefulness of our proposed method, we acquired the 3D shape of a miniature bunny model produced using micro-stereolithography based on the bottom-up system. Figure 4 compares silhouette images obtained using UV and visible light. In these experiments, the transparent bunny model made from the epoxy-based resin was used as the target object (Figure 4a). To obtain a silhouette image with visible light, we replaced the UV LED with a halogen fiber light source (LG-PS2, Olympus Corp., Tokyo, Japan) using the optical setup shown in Figure 2. As shown in Figure 4b, the resulting silhouette image was a blocky, gray photograph caused by transmission and refraction from the transparent object. It was not suitable for obtaining a correct binarized silhouette image without artificial voids. On the other hand, the silhouette image obtained using UV transmitted illumination was a substantially uniform, black photograph because most of the UV light was absorbed. Therefore, a correct silhouette of the transparent target object could be obtained after proper binarization of the silhouette image. This shows the advantage of using UV transmitted illumination for capturing correct, high-contrast silhouette images.

**Figure 5:**
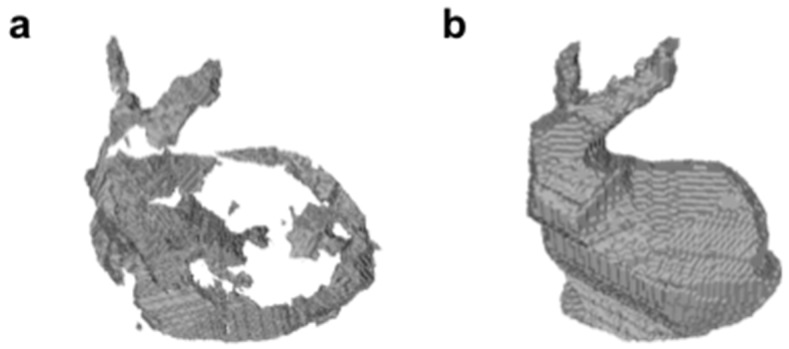
3D shape reconstruction of a bunny model using (**a**) visible light and (**b**) UV transmitted light illumination.

The 3D shape of the bunny model was reconstructed using the SFS method with visible and UV light. Figure 5 shows the reconstructed triangular mesh models. As Figure 5a shows, some portions of the bunny model could not be reconstructed using visible light. On the other hand, all portions of the bunny model were completely reconstructed using UV light. This was because the use of high-contrast silhouette images with slightly uneven brightness levels made it possible to obtain the correct visual hull of the target object.

### 3.3. Evaluating the Accuracy of 3D Shape Acquisition Using the Pillar Array Model

**Figure 6:**
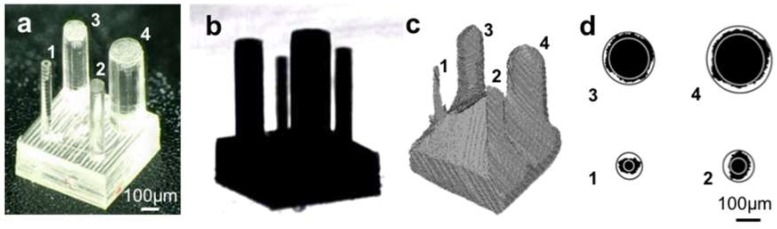
3D shape reconstruction of a 3D printed pillar array model made of acrylate-based resin. (**a**) optical microscope image; (**b**) silhouette obtained using UV illumination; (**c**) reconstruction of the pillar array model using a shape from silhouette method; and (**d**) a cross section at half the height of each reconstructed pillar.

To evaluate the accuracy of the 3D shape acquisition system, a pillar array model (Figure 6a) containing pillars of different diameters was fabricated using micro-stereolithography based on the top-down system. The diameter of each of the actual pillars was measured using an optical microscope; these results are summarized in Table 1. Figure 6b shows the silhouette image of the pillar array model captured using UV transmitted illumination. All of the pillars were captured with high contrast. The 3D shape of the pillar array model was reconstructed using multiple silhouette images taken from different viewpoints (Figure 6c). Although the smallest pillar was slightly distorted, all the pillars were reconstructed. The average diameter of each of the reconstructed pillars was calculated using a cross section at half the height of each pillar. In this calculation, we used the average of an inscribed circle and a circumscribed circle for each pillar, as shown in Figure 6c. The averaged diameters of these reconstructed pillars are summarized in Table 1. In these results, the difference between the actual and averaged diameters of the reconstructed pillars ranged from 1–20 µm.

There are some parameters that could be used to reduce the errors in the reconstructed 3D shape. In the SFS method, we used a pixel size of 11.7 µm to represent the voxel-based model. The reconstructed 3D model could be smoother and finer if a smaller voxel size was used. The number of CCD camera elements also affected the quality of the silhouette images. Using a higher resolution camera could also reduce the minimum pixel size of the silhouette images and make the visual cone more precise. Additionally, the magnification and depth of field of the imaging lens are important parameters. Since there is a trade-off relationship between the magnification and depth of field, observing 3D microscopic objects using an optical microscope is an intrinsic problem. As shown in Figure 6b, the silhouette image of the pillars has a high contrast but the focus is blurry because of the limited depth of field. To overcome these problems, we could use an image fusion technique for a sequence of images taken by changing the position of the focus along the optical axis. This technique would provide sharp silhouettes even under a high magnification [19].

## 4. Conclusions

We demonstrated a simple and low-cost 3D shape acquisition method for transparent 3D printed microscopic objects. This method employed highly UV-absorbent 3D printed polymer objects to obtain high-contrast silhouette images of transparent 3D objects using UV transmitted illumination. Multiple silhouette images taken from different viewpoints made it possible to reconstruct the 3D shapes of the transparent 3D printed objects using the SFS method, with a 3D shape acquisition system constructed using a UV LED, a CCD camera and a rotation stage. A bunny model as small as 1 mm was successfully reconstructed with this system using an imaging lens with a 1× magnification. By changing the imaging lens, this system could be applicable to macro- and micro-scale models. In addition, transparent 3D printed models made from glass as well as polymer [20,21] could be observed using this method. Therefore, this method could be an inexpensive and useful tool for a 3D scanner and a way to inspect the appearance of transparent 3D objects without the need for time-consuming pre- and post-processing techniques.

## Figures and Tables

**Table 1:** Diameter of each pillar of a 3D printed pillar array model.

Pillar Number	Actual Pillar Diameter Measured by an Optical Microscope	Averaged Pillar Diameter Estimated by SFS Method
1	106 μm	94 μm
2	152 μm	151 μm
3	248 μm	268 μm
4	347 μm	333 μm
